# The impact of Dual Eligible Special Need Plan regulations on healthcare utilization

**DOI:** 10.1186/s12913-021-06228-3

**Published:** 2021-03-07

**Authors:** Kimberly Danae Cauley Narain, Jessica Harwood, Carol Mangione, O. Kenrik Duru, Susan Ettner

**Affiliations:** 1 Division of General Internal Medicine and Health Services Research (GIM/HSR), Department of Medicine, University of California Los Angeles, 1100 Glendon Ave., Suite 850, Los Angeles, CA 90024 USA; 2 Center for Health Advancement, Fielding School of Public Health, University of California Los Angeles, 650 Charles Young Dr., 31-269 CHS, Box 951772, Los Angeles, CA 90095-1772 USA; 3 Health Policy and Management, Fielding School of Public Health, UCLA, 650 Charles Young Dr. S., 31-269 CHS, Box 951772, Los Angeles, CA 90095-1772 USA

**Keywords:** Dual-eligible beneficiaries, Healthcare utilization, Medicare

## Abstract

**Background:**

To determine if requiring Dual Eligible Special Need Plans (D-SNPs) to receive approval from the National Committee of Quality Assurance and contract with state Medicaid agencies impacts healthcare utilization.

**Methods:**

We use a Multiple Interrupted Time Series to examine the association of D-SNP regulations with dichotomized measures of emergency room (ER) and hospital utilization. Our treatment group is elderly D-SNP enrollees. Our comparison group is near-elderly (ages 60–64) beneficiaries enrolled in Medicaid Managed Care plans (*N* = 360,405). We use segmented regression models to estimate changes in the time-trend and slope of the outcomes associated with D-SNP regulations, during the post-implementation (2012–2015) period, relative to the pre-implementation (2010–2011) period. Models include a treatment-status indicator, a monthly time-trend, indicators and splines for the post-period and the interactions between these variables. We conduct the following sensitivity analyses: (1) Re-estimating models stratified by state (2) Estimating models including interactions of D-SNP implementation variables with comorbidity count to assess for differential D-SNP regulation effects across comorbidity level. (3) Re-estimating the models stratifying by race/ethnicity and (4) Including a transition period (2012–2013) in the model.

**Results:**

We do not find any statistically significant changes in ER or hospital utilization associated with D-SNP regulation implementation in the broad D-SNP population or among specific racial/ethnic groups; however, we do find a reduction in hospitalizations associated with D-SNP regulations in New Jersey (DD level = − 3.37%; *p* = 0.02)/(DD slope = − 0.23%; *p* = 0.01) and among individuals with higher, relative to lower levels of co-morbidity (DDD slope = − 0.06%; p = 0.01).

**Conclusions:**

These findings suggest that the impact of D-SNP regulations varies by state. Additionally, D-SNP regulations may be particularly effective in reducing hospital utilization among beneficiaries with high levels of co-morbidity.

**Supplementary Information:**

The online version contains supplementary material available at 10.1186/s12913-021-06228-3.

## Background

More than 10 million individuals referred to as dual-eligible beneficiaries receive both Medicare and Medicaid health insurance benefits as a consequence of low income and assets in conjunction with age ≥ 65 years or disability [1]. Relative to Medicare-only beneficiaries, dual-eligible beneficiaries have a higher chronic disease burden, more functional impairments and more behavioral health conditions. Consequently, dual-eligible beneficiaries have higher levels of healthcare utilization than Medicare-only beneficiaries and account for a disproportionate share of both Medicare and Medicaid program spending. In 2013 dual-eligible beneficiaries were only 20% of the Medicare population but accounted for 34% of total program spending. Similarly, they made up 15% of Medicaid enrollment but generated 32% of total program costs [1].

While differences in need partially explain the higher health care utilization and costs among dual-eligible beneficiaries relative to Medicare-only beneficiaries, care fragmentation has also been shown to play a role [2]. A high level of care fragmentation stems from receiving health care through both Medicare and Medicaid, separate programs with distinct funding streams, eligibility criteria, administrative processes, medical providers, covered services and accountability mechanisms [3]. Specifically, Medicare is a purely federal program which covers most acute care services such as inpatient and outpatient care, physician services, diagnostic and preventive care and since 2006, prescription medications. Medicaid, which covers Medicare premiums, patient cost-sharing requirements, and long-term services and supports such as home health and nursing home care, is operated by states with federal oversight and paid for with a mix of federal and state funding [4]. Medicaid and Medicare have traditionally had little incentive to coordinate care for dual-eligible beneficiaries. For example, Medicare has had little incentive to keep dual-eligible beneficiaries out of nursing homes since the costs would be borne primarily by states. Likewise, states have had little incentive to reduce hospitalizations among dual-eligible beneficiaries because hospitalization costs would be mainly absorbed by Medicare.

In 2003, the Medicare Modernization Act authorized Dual-Eligible Special Needs Plans (D-SNPs), managed care plans offered by private insurers and targeted specifically to dual-eligible beneficiaries, with the goal of aligning financial incentives across Medicare and Medicaid to promote care coordination and reduce costs. Insurers offering D-SNPs are paid a fixed monthly amount per person (capitated payment) by Medicare to provide the full range of Medicare services and coordinate Medicaid services among dual-eligible beneficiaries. D-SNPs are at financial risk for Medicare costs in excess of the capitated payment but they are permitted to retain a portion of the Medicare payment not spent on covered services for contractually mandated activities [4]. When D-SNPs were initially authorized, there was no requirement to provide Medicaid benefits to beneficiaries and no specific Model of Care requirements (MOC). Consequently, the majority of D-SNPs did not have any formal relationship with state Medicaid agencies and did not provide a coordinated Medicare-Medicaid product [5]. Between 2006 and 2017 the proportion of dual-eligible beneficiaries enrolled in managed care plans grew from 12 to 32% [6]. In 2017, 2,060,218 dual-eligible beneficiaries were enrolled in D-SNPs [7].

Since the original authorization of D-SNPs, Congress has passed additional legislation with the aim of increasing quality of care, improving health outcomes and reducing healthcare costs among dual-eligible beneficiaries enrolled in D-SNPs [8]. The Medicare Improvement Act for Patients and Providers (MIPPA) of 2008, as amended by the Patient Protection and Affordable Care Act (PP-ACA) of 2010, required all D-SNPs to have a contract with states in which they operated and specified minimum requirements for these contracts. The PP-ACA mandated that as of 2012, any new D-SNP would have to either provide Medicaid benefits in their capitated benefit package or arrange for Medicaid benefits to be provided through either a Medicaid Managed Care (MMC) plan or Medicaid FFS, depending on the requirements of the state [9]. In 2013, this regulation became binding for both new and old D-SNPs. The PP-ACA also required that D-SNPs receive approval on their MOC from the National Committee of Quality Assurance (NCQA) in order to operate [10].

## New contribution

No studies, to our knowledge, have evaluated the impact of the PP-ACA D-SNP regulations (hereinafter referenced as “D-SNP regulations”) on healthcare utilization among dual-eligible beneficiaries. The only multi-state study of the effects of D-SNPs covered the pre-regulation time period (2007–2011) and focused exclusively on healthcare expenditures. Zhang et al. (2018) examined the impact of D-SNP penetration on state Medicare, Medicaid and total healthcare spending [11]. Controlling for state-level demographic, socioeconomic, and healthcare resource factors along with state and year fixed-effects, having a higher percentage of dual-eligible beneficiaries enrolled in D-SNPs was linked with reduced Medicare spending per enrollee but not reductions in Medicaid or total healthcare spending. Examining the effects of PP-ACA regulation on healthcare utilization among dual-eligible beneficiaries enrolled in D-SNPs is important, as enrollment in these plans is being incentivized through mechanisms such as seamless conversion (default enrollment of MMC beneficiaries into D-SNPs when they age into Medicare). Furthermore, the benefit of D-SNPs over other coverage options such as fee-for-service Medicare and Medicare Advantage Plans has not been clearly demonstrated [12].

In addition to examining effects of D-SNP regulations among the overall dual-eligible population, we conduct state-stratified analysis to reflect the heterogeneity in D-SNP regulation implementation across states. It is also important to examine effects across various subpopulations of beneficiaries. It is possible that certain subpopulations may experience more benefits associated with D-SNP enrollment than others. For example, the evidence supports the benefits of enhanced care coordination practices among subpopulations with the highest levels of medical complexity [13]. Consequently, D-SNPs would be expected to have their most beneficial effects among the subset of the dual-eligible population with the highest level of co-morbidity.

Evidence has also shown that racial/ethnic minority beneficiaries, which constitute a disproportionate share of dual-eligible beneficiaries, are often enrolled in lower-performing managed care plans relative to non-minority beneficiaries [14]. Consequently, augmented D-SNP care coordination and MOC requirements may have stronger beneficial effects on health outcomes among racial/ethnic minorities who experience significant health disparities.

This study contributes to the literature by using a quasi-experimental study design and administrative claims data from three states (Arizona, New Jersey, Tennessee), from one of the largest insurers in the United States, to examine the impact of PP-ACA D-SNP regulations on emergency room (ER) and hospital utilization among community-dwelling elderly D-SNP enrollees, across states, co-morbidity level and racial/ethnic minority status. We hypothesize the following: 1) D-SNP regulations will lead to reduced ER and hospital utilization among D-SNP enrollees; 2) The effect of D-SNP regulations will be larger among enrollees with more, relative to less co-morbidity and 3) The D-SNP regulation effects will be stronger among racial/ethnic minorities.

## Conceptual framework

The study examines the ER/hospital utilization effects PP-ACA D-SNP regulations, specifically Medicaid contracting and NCQA MOC requirements. D-SNP contracting requirements grant state Medicaid programs the authority to define the scope of Medicaid benefits that a D-SNP must cover or coordinate, potentially shifting the financial risk for Medicaid cost-sharing requirements and high-cost Medicaid services such as nursing home care to the D-SNPs or the insurers that offer them. For example, states can mandate that insurers offering D-SNPs have a companion Medicaid Managed Long Term Services and Supports (MLTSS) plan, an insurance plan that offers Medicaid covered services under a capitated payment arrangement. This increased financial exposure at the plan/insurer level may incentivize better care coordination among D-SNPs, leading to reduced ER and hospital utilization. During the time period covered by this analysis, all three states had implemented contract requirements mandating closer relationships between D-SNPs and the entity offering MLTSS services. Specifically, Arizona, only contracted with D-SNPs that had a companion MLTSS plan, New Jersey restricted D-SNP enrollment to beneficiaries covered by an aligned Managed Care Organization (MCO) and Tennessee required D-SNPs formed after 2014 to have a companion MLTSS plan [15].

The NCQA MOC requirements may also have the effect of improving care coordination among D-SNP beneficiaries. MOC requirements such as maintaining network-level medical provider access, the use of interdisciplinary care teams, performance of routine patient assessment, care management and performance measurement are aimed at developing a comprehensive assessment of beneficiary needs and facilitating the timely match between patient care needs and services. For example, a comprehensive patient assessment, conducted by a multi-disciplinary team (doctors, nurses, pharmacist, social workers), may reveal the need for home health services that may prevent a patient from going to the ER or requiring hospitalization [16]. During the time period covered by this analysis all three states had contract specifications that reflected NCQA MOC requirements. For instance, Arizona mandated the presence of a contact person at each plan, tasked with sharing information to facilitate coordination of behavioral health, disease management and case management when benefits switched from Medicare to Medicaid coverage. New Jersey also mandated integrated care management in it’s contracts. Lastly, Tennessee required coordination with the Medicaid MCO for discharge planning; including ensuring MLTSS were offered in the most integrated and cost-effective setting [15].

## Methods

### Overview of study design

**Fig. 1:**
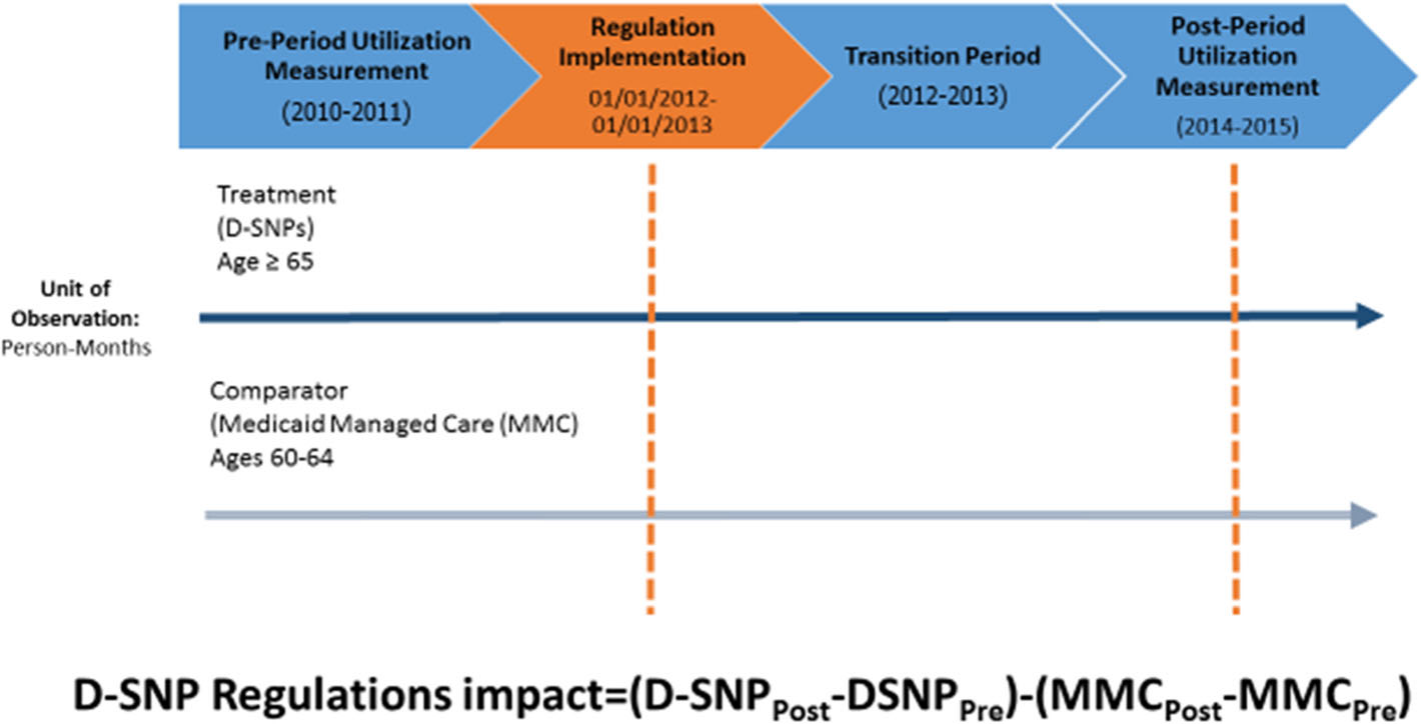
Study Design Overview

An individual-level, Multiple-group Interrupted Time Series (MITS) study design was used to estimate changes in the monthly time trend of ER and hospital utilization associated with the D-SNP regulations, between the pre-implementation period (2010–2011) and the post-implementation period (2012–2015) (Fig. 1) [17]. The MITS is a strong study design in that it can provide valid effect estimates even if the treatment and comparison groups have different baseline intercepts and slopes for the outcomes of interest. The MITS assumes that the comparison group is not impacted by the intervention and that the pre-post intervention changes in the intercept and slope of the outcome would not have differed across the treatment and comparison groups, in the absence of the intervention. The treatment group is elderly community-dwelling D-SNP enrollees and the comparison group is near-elderly MMC enrollees. The use of MMC enrollees as a comparison group is advantageous because it may help net out the healthcare utilization effects of temporally proximate changes in MMC plans which could potentially confound the effect estimates for D-SNP regulations.

### Data and study cohort

The study data was obtained from UnitedHealthcare (UHC), the largest health insurance provider in the United States. As of 2018, UnitedHealthcare had the largest market share for D-SNPs at 31% [18]. Our data set includes 1) medical and behavioral health insurance claims, providing information regarding utilization and diagnosis; 2) enrollment eligibility information; and 3) demographic information. The study cohort consists of community-dwelling individuals, enrolled in either a D-SNP plan (age ≥ 65 years) or MMC plan (age ≥ 60–64 years), in one of 3 states (Arizona, New Jersey, Tennessee). The study population is limited to the above-mentioned three states because they are the only states in the sample with substantial numbers of D-SNP enrollees both before and after implementation of D-SNP regulations. We identify D-SNP and MMC beneficiaries using information on the UnitedHealthcare insurance product through which their benefits are received (eligibility data). Insurance benefit program codes covering dual-eligible beneficiaries are retained in the sample as well as program codes covering Medicaid beneficiaries receiving Temporary Assistance for Needy Families and Medicaid beneficiaries receiving Supplemental Security Income (SSI). We further limit the sample to individuals with at least 7 months of continuous enrollment (the first 6 months are used to define covariates and subsequent months are used to measure outcomes), individuals of Non-Hispanic Black, Non-Hispanic White or Hispanic race/ethnicity and individuals not receiving dialysis. The unit-of-analysis is the person-month and we have a total of 360,405 person months in the study, representing 16,396 unique individuals. The sample size flow chart is shown in Table 1.

**Table 1:** Sample Size Flowchart

	N (person-months)	% retained from previous N
People aged 60+ living in NJ, TN, or AZ	3,078,591	100.0%
And race/ethnicity is Non-Hispanic White, Non-Hispanic Black, or Hispanic	1,779,655	57.8%
And included in Treatment group (dual-eligibles 65+ enrolled in D-SNP) or Control group (individuals 60–64 enrolled in Medicaid Managed Care [TANF & SSI without Medicare] but not dual-eligible)	591,834	33.3%
And not on dialysis	587,650	99.3%
And limit to people with at least 7 months of continuous enrollment, with the seventh month and after included in the sample	450,323	76.6%
Years 2010–2015	360,405	80.0%

### Outcomes

Our outcomes included dichotomous indicators for whether an enrollee had any hospital admissions and any ER visits in the given month.

### D-SNP regulations

Figure 1 provides an overview of the study design. Specifically, we identify the effect of D-SNP regulations using the equation below.

$$ {Utilization}_{it}={\beta}_{0 it}+{\beta}_1\left({T}_t\right)+{\beta}_2\left({Regulation}_t\right)+{\beta}_3\left( Time\ {After\ Regulation}_t\right)+{\beta}_4\left({DSNP}_i\right)+{\beta}_5\left({DSNP}_i\right)\left({T}_t\right)+{\beta}_6\left({DSNP}_i\right)\left({Regulation}_t\right)+{\beta}_7\left({DSNP}_i\right)\left( Time\ {After\ Regulation}_t\right)+{\varepsilon}_{it} $$

Where *Utilization*_*it*_ is the utilization (emergency room/hospital) of person *i* in month *t*. “T” is a monthly linear time trend of utilization. “Regulation” is an indicator coded as “1” if the data is from a year ≥2012 and coded as “0” otherwise. “Time After Regulation” is a spline variable counting months from January 2012 forward (January 2012 = 1, February 2012 = 2, etc.; months from a year < 2012 are coded as “0”). “D-SNP” is an indicator coded as “1” if the data is from a D-SNP beneficiary and coded as “0” if the data is from a MMC beneficiary. *β*_0*it*_ is the baseline healthcare utilization for the month in the near-elderly MMC beneficiary group, during the pre-regulation period. *β*_1_ is the slope of utilization among the MMC beneficiary group during the pre-regulation period. *β*_2_ is the immediate change in the level of utilization among the MMC beneficiary group immediately after implementation of D-SNP regulations. *β*_3_ is the gradual change in the slope of utilization among the MMC beneficiary group after regulation implementation. *β*_4_ is the difference in the pre-regulation utilization level across the MMC and D-SNP beneficiary groups. *β*_5_ is the pre-regulation difference in the utilization slopes across the MMC and D-SNP beneficiary groups. *β*_6_ is the difference in the size of the pre-post regulation change in the level of utilization across the MMC and D-SNP beneficiary groups. *β*_7_ is the difference in the size of the pre-post regulation change in the slope of utilization across the MMC and D-SNP beneficiary groups. *β*_6_ and *β*_7_ are the coefficients of interest and reflect the impact of D-SNP regulations net of secular time trends, provided the assumptions of the MITS are met. Coefficients for *β*_6_ and *β*_7_ that are statistically significant and ≤ 0 are supportive of our first hypothesis.

### Other covariates

Our other covariates (not shown in the equation above) include demographic factors (sex, age, race/ethnicity), behavioral health conditions (depression, schizophrenia and substance abuse), medical co-morbidities (hypertension, hyperlipidemia, diabetes, myocardial infarction, congestive heart failure, stroke, atrial fibrillation, chronic kidney disease, chronic obstructive pulmonary disease, asthma, liver disease, cancer, HIV, arthritis and dementia), SSI receipt and state fixed-effects.

### Statistical analysis

For each outcome, we model the impact of D-SNP regulations using a Linear Probability Model (Ordinary Least Squares Regression applied to dichotomous outcomes). Use of the LPM model allows for direct interpretation of the regression coefficients and if the sample size is large, as is the case for this study, the point estimates will be statistically consistent (although not minimum-variance). Standard errors were adjusted for within-person clustering of months using generalized estimating equations [19]. To investigate the impact of D-SNP regulations across states, we conduct state-stratified analyses. To assess the prospect of differential impacts of D-SNP regulations across levels of co-morbidity, we run models interacting patient co-morbidity count with variables capturing the implementation of D-SNP regulations. To evaluate for differential D-SNP regulation effects across race/ethnicity group, we run models stratified by race/ethnicity (Non-Hispanic Black vs. Non-Hispanic White vs. Hispanic). Additionally, we construct models that include interactions between variables capturing D-SNP regulation implementation and each racial/ethnic category to ascertain if any potential differences observed in the race-ethnicity stratified models are statistically significant. Lastly, we conduct a sensitivity analysis of the models evaluating the effect of D-SNP regulations on all D-SNP beneficiaries by constructing models that include a two-year transition period (2012–2013) before the full effect is observed. The transition period is incorporated into the model by including an indicator coded as “1” if the data is from years 2012–2013 and coded as “0” otherwise. Commensurately, we change the coding of our “D-SNP Regulation” variable to “1” if the data is from years 2014–2015 and “0” otherwise. We also include a transition period spline variable (counting months in 2012–2013, from 1 to 24; coded as “0” otherwise). We change the coding of the “Time After Regulation” variable to count months from 2014 to 2015 (coded “0” otherwise). Finally, we interact the “transition” indicator and spline variables with our “D-SNP” variable to examine the impact of D-SNP regulations on the level and slope of utilization during the transition period, respectively.

## Results

The person-month demographic and clinical characteristics of the D-SNP and MMC-enrolled beneficiaries can be found in Table 2. In addition to containing more months from younger enrollees, the comparison sample included more months from Non-Hispanic Blacks, fewer months from Hispanics, fewer months from females and more months from individuals with higher levels of co-morbidity. The proportion of months with any ER visit and any hospital utilization was also higher in the comparison sample (6% vs. 3%) and (3% vs. 2%), respectively. The *p*-value for all above-mentioned differences was < 0.01.

**Table 2:** Demographic and clinical characteristics of D-SNP and Medicaid Managed Care enrollees, averaged over the Pre Period (2010–2011)

N	Medicaid Managed Care		D-SNP		P-Value^1^	
	59,618		25,492			
	n	%	n	%		
Mean age	62		73		0.00	*
Race/ethnicity						
Non-Hispanic White	35,775	60	12,536	49	0.00	*
Non-Hispanic Black	20,641	35	3342	13	0.00	*
Hispanic	3202	5	9614	38	0.00	*
Female	33,999	57	17,826	70	0.00	*
Any hospitalization	1569	3	535	2	0.01	*
Any emergency department visit	3338	6	854	3	0.00	*
Calendar year						
2010	28,014	47	11,039	43	0.00	*
2011	31,604	53	14,453	57	0.00	*
State of residence						
Arizona	8248	14	22,253	87	0.00	*
New Jersey	6297	11	2024	8	0.01	*
Tennessee	45,073	76	1215	5	0.00	*
Medicaid category						
TANF	4014	7	0	0		
SSI (without Medicare)	55,604	93	0	0		
Medicare/Medicaid dual	0	0	25,492	100		
Hypertension	23,150	39	7139	28	0.00	*
Depression	5550	9	876	3	0.00	*
Atrial Fibrillation	3801	6	1439	6	0.35	
Hyperlipidemia	15,039	25	5451	21	0.01	*
Arthritis	6750	11	2390	9	0.06	
Substance Abuse	6166	10	574	2	0.00	*
Myocardial Infarction	5299	9	1945	8	0.17	
Congestive Heart Failure	2892	5	554	2	0.00	*
Chronic Obstructive Pulmonary Disease	8144	14	1649	6	0.00	*
Asthma	3114	5	824	3	0.00	*
Chronic Kidney Disease (Stage IV or V)	281	0	187	1	0.27	
Liver Disease	1360	2	273	1	0.01	*
N	Medicaid Managed Care	D-SNP	P-Value^1^			
	59,618		25,492			
	n	%	n	%		
Dementia	867	1	455	2	0.40	
Cancer	4323	7	1344	5	0.02	*
HIV	265	0	16	0	0.06	
Diabetes	18,704	31	9204	36	0.00	*
Stroke	3486	6	1215	5	0.15	
Schizophrenia	2760	5	249	1	0.00	*
Mean comorbidity count	2		1		0.00	*
Pre, Transition and Post:						
Total person-months of data in the analyses	212,970		147,435			
Total unique people in the analyses	10,606		5790			

^1^. *P*-values are from regressions controlling for clustering at the person level. All regressions were logistic regressions except in comparing age and the comorbidity count between treatment groups, which used gamma regression. *p < 0.05

**Fig. 2:**
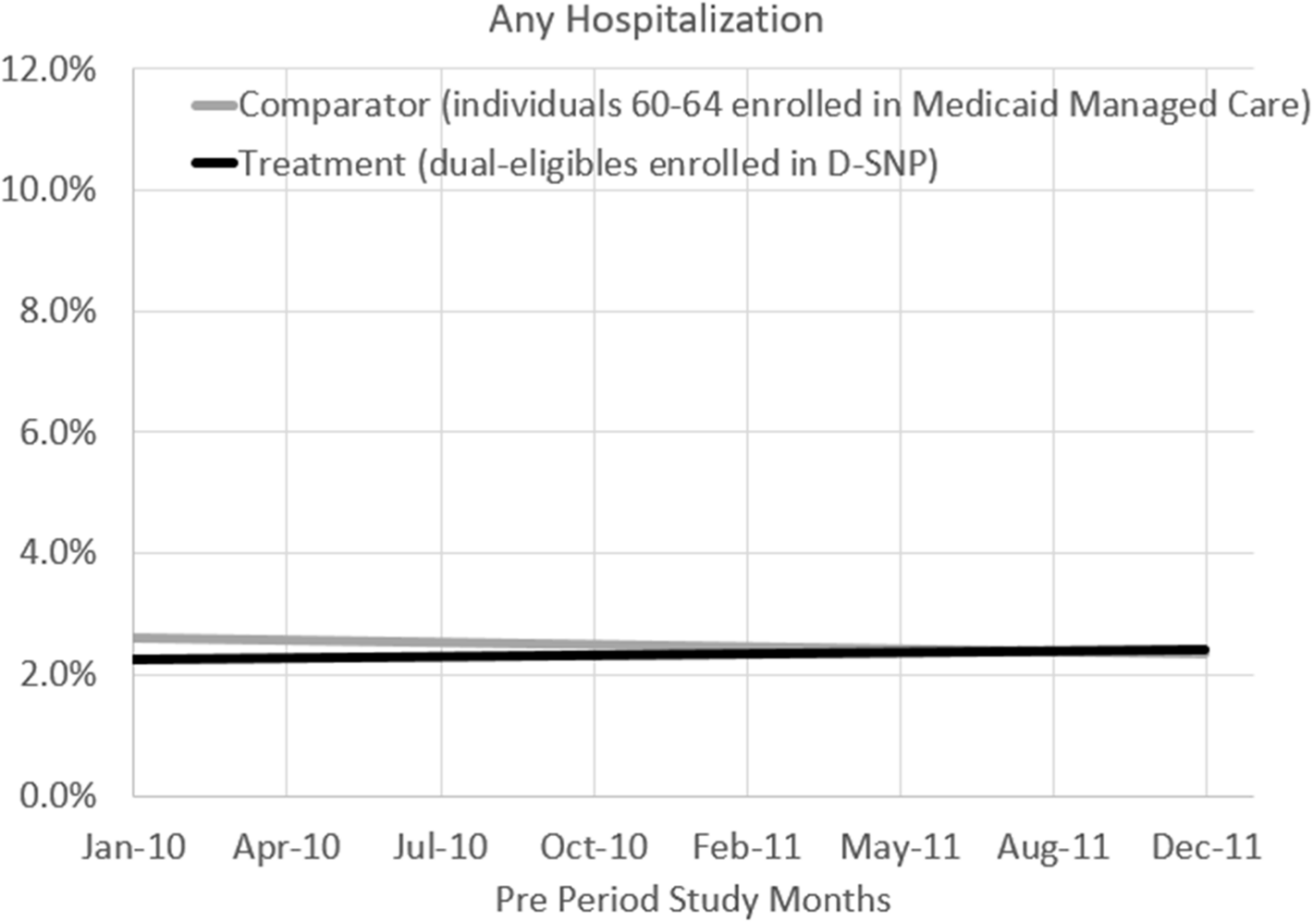
Pre-period Hospitalization Trends

**Fig. 3:**
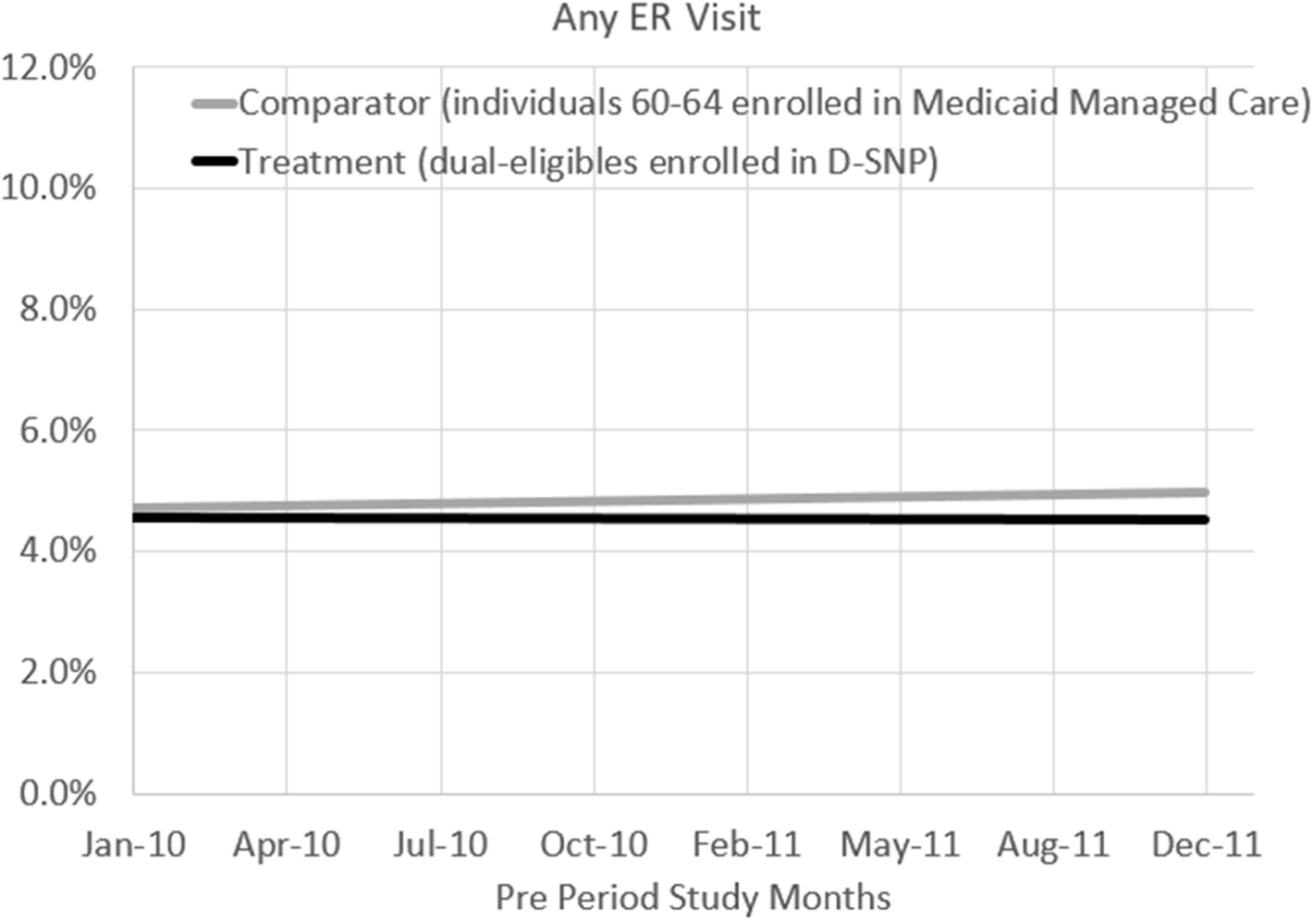
Pre-period Emergency Room Visit Trends

For completeness, we compare secular time trends for ER/hospital utilization across the D-SNP and the MMC groups during the pre-regulation period and do not detect significant differences (2010–2011) (Figs. 2 and 3).

In our adjusted analyses, shown in Table 3, we did not find any statistically significant changes in ER or hospital utilization associated with the implementation of D-SNP regulations, among the total population. However, in state-stratified analyses, D-SNP regulation implementation was negatively associated with hospitalizations in New Jersey (DD level = − 3.37%; *p* = 0.02)/(DD slope = − 0.23%; *p* = 0.01); (Table 4). Among the models interacting D-SNP regulation implementation with co-morbidity count, only the model of hospital utilization showed a decline in utilization associated with D-SNP regulation implementation (DDD slope = − 0.06%; p = 0.01); (Table 5). This estimate represents the additional impact of D-SNP regulation implementation on hospitalization for each one-unit increase in co-morbidity count. None of the race/ethnicity-stratified models show significant impacts of D-SNP regulations on ER/hospital utilization (Table 6). Likewise, none of the interaction terms capturing differential effects of D-SNP regulation implementation across race/ethnicity groups were statistically significant (not shown). Lastly, the sensitivity analysis including transition period variables did not show any effects of D-SNP regulations on healthcare utilization (Supplementary Table 1).

**Table 3:** Interrupted time series (ITS) segmented regression analysis: difference-in-difference (DID) estimates, comparing treatment (dual-eligibles enrolled in D-SNP) vs. comparator (individuals 60–64 enrolled in Medicaid Managed Care) on changes in monthly utilization time trends associated with DSNP Regulations

		Post Period (vs. Pre Period)			
Sample	Outcome	DID Level^**A**^	P-Value	DID Slope^**B**^	P-Value
Total^1^	Any Hospitalization	−0.41%	0.19	−0.02%	0.39
	Any ED Visit	−0.70%	0.07	0.00%	0.90

Notes: Linear regression used for utilization outcomes. Sample is person-months from 2010 to 2015. Regression covariates include Group (treatment vs. comparator); a linear monthly time trend, indicators and splines for the post period (2012–2015); and the interactions between these variables. Other covariates included sex, age, race (full sample model only), indicator for SSI status, 18 comorbidity indicators, and state fixed effects. Repeated measures adjusted for using Huber-White robust standard errors, clustering at the person level. ^1^. N = 360,405

^A^. Difference between treatment vs. comparator in the discontinuity (change in level) for the post period, measured using the interaction between Group & an indicator variable for the post period

^B^. Difference between treatment vs. comparator in the change in slope for the post period, measured using the interaction between Group & a spline variable for the post period

**Table 4:** Interrupted time series (ITS) segmented regression analysis: difference-in-difference (DID) estimates, comparing treatment (dual eligibles enrolled in D-SNP) vs. comparator (individuals 60–64 enrolled in Medicaid Managed Care) on changes in monthly utilization time trends associated with ACA DSNP Changes

		Post Period (vs. Pre Period)			
State	Outcome	DID Level^1^	*P*-Value	DID Slope^2^	P-Value
AZ	Any Hospitalization	− 0.19%	0.702	0.01%	0.710
	Any ED Visit	−0.48%	0.461	−0.03%	0.545
NJ	Any Hospitalization	−3.37%	0.022*	−0.23%	0.014*
	Any ED Visit	−2.87%	0.057	−0.18%	0.103
TN	Any Hospitalization	0.32%	0.565	−0.01%	0.903
	Any ED Visit	−1.38%	0.156	0.05%	0.562

Notes: Linear regression used for utilization outcomes. Sample is person-months from 2010 to 2015. *denotes significance at p < .05. Regression covariates of interest were study Group (treatment vs. comparator); a linear monthly time trend, indicators and splines for the post period (2012–2015); and the interactions between these variables and Group. Other covariates included sex, age, race, indicator for SSI status, and 18 comorbidity indicators. Repeated measures adjusted for using Huber-White robust standard errors, clustering at the person level

^1^. Difference between treatment vs. comparator in the discontinuity (change in level) for the post period, measured using the interaction between Group & an indicator variable for the post period

^2^. Difference between treatment vs. comparator in the change in slope for the post period, measured using the interaction between Group & a spline variable for the post period

**Table 5:** Interrupted time series (ITS) segmented regression analysis: difference-in-difference-in-difference (DDD) estimates, comparing treatment (dual-eligibles enrolled in D-SNP) vs. comparator (individuals 60–64 enrolled in Medicaid Managed Care) on changes in monthly utilization time trends associated with DSNP Regulations by Co-morbidity Count

		Post Period (vs. Pre Period)			
		DDD Level^**A**^	P-Value	DDD Slope^**B**^	P-Value
Total Sample	Any Hospitalization	− 0.12%	0.67	− 0.06%	0.01*
	Any ED Visit	−0.16%	0.63	−0.04%	0.12

Notes: Linear regression used for utilization outcomes. Sample is person-months from 2010 to 2015. *denotes significance at p < .05. Regression covariates include Group (treatment vs. comparator); a linear monthly time trend, indicators and splines for the post period (2012–2015), a continuous variable capturing the number of co-morbidities; and the interactions between these variables. Other covariates included sex, age, race, indicator for SSI status, 18 comorbidity indicators, and state fixed effects. Repeated measures adjusted for using Huber-White robust standard errors, clustering at the person level. N = 360,405

^A^. How the difference between treatment vs. comparator in the discontinuity (change in level) for the post period changes for a one-unit increase in co-morbidity count, measured using the interaction between Group, comorbidity count & an indicator variable for the post period

^B^. How the difference between treatment vs. comparator in the change in slope for the post period changes for a one-unit increase in co-morbidity count, measured using the interaction between Group, comorbidity count & a spline variable for the post period

**Table 6:** Interrupted time series (ITS) segmented regression analysis: difference-in-difference (DID) estimates, comparing treatment (dual-eligibles enrolled in D-SNP) vs. comparator (individuals 60–64 enrolled in Medicaid Managed Care) on changes in monthly utilization time trends associated with DSNP Regulations, Across Racial/ethnic Group

		Post Period (vs. Pre Period)			
Sample	Outcome	DID Level^**A**^	***P***-Value	DID Slope^**B**^	P-Value
Non-Hispanic White^1^	Any Hospitalization	−0.45%	0.28	−0.04%	0.22
	Any ED Visit	−0.21%	0.69	−0.01%	0.89
Non-Hispanic Black2^2^	Any Hospitalization	−0.70%	0.37	0.01%	0.87
	Any ED Visit	−1.53%	0.10	−0.00%	0.99
Hispanic^3^	Any Hospitalization	−1.10%	0.14	−0.01%	0.84
	Any ED Visit	−0.96%	0.33	0.02%	0.73

Notes: Linear regression used for utilization outcomes. Sample is person-months from 2010 to 2015. Regression covariates include Group (treatment vs. comparator); a linear monthly time trend, indicators and splines for the post period (2012–2015); and the interactions between these variables. Other covariates included sex, age, indicator for SSI status, 18 comorbidity indicators, and state fixed effects. Repeated measures adjusted for using Huber-White robust standard errors, clustering at the person level. ^1^. *N* = 205,371 ^2^. *N* = 90,506 ^3^. *N* = 64,528

^A^. Difference between treatment vs. comparator in the discontinuity (change in level) for the post period, measured using the interaction between Group & an indicator variable for the post period

^B^. Difference between treatment vs. comparator in the change in slope for the post period, measured using the interaction between Group & a spline variable for the post period

## Discussion

We conducted the first study analyzing the impact of PP-ACA D-SNP regulations on ER and hospital utilization using administrative data from one of the largest health insurers in the United States, for three states (Arizona, Tennessee and New Jersey) with a MITS study design. We did not find any statistically significant effects of D-SNP regulations among the total population of D-SNP enrollees, which is inconsistent with our hypothesis. However, we did find reductions in hospitalizations associated with D-SNP regulation implementation in New Jersey which supports our hypotheses. Additionally, we found evidence that the impact of D-SNP regulations on hospitalization grows as the number of co-morbidities increases, which supports our hypothesis that D-SNP regulations would have stronger effects among the subpopulation with the highest levels of co-morbidity. We also do not find evidence of differential impacts of D-SNP regulations across racial/ethnic subgroups, which does not support our hypothesis of stronger D-SNP regulation utilization effects among racial/ethnic minorities.

The inability of this study to detect utilization effects of D-SNP regulations among the total population of D-SNP enrollees may be attributable to the states included in the study sample. Specifically, Arizona, which contributes the bulk of the data for D-SNP enrollees, developed the Arizona Long Term Care System (ALTCS), a managed care plan for dual-eligible beneficiaries (in the community or long term care settings), requiring nursing home level care (developmental disability or elderly with a physical disability) in 1989 [20]. D-SNP enrollment in Arizona is restricted individuals who are enrolled in the ALTCS [21]. This particular subset of the population may not be reflective of the broader population of dual-eligible beneficiaries in terms of disability and co-morbidity status. Specifically, our descriptive statistics show lower baseline levels of co-morbidity and ER/hospital utilization among D-SNP enrollees, relative to our MMC enrollees. Additionally, Arizona has long-standing experience with delivering health care in a managed setting and care integration. Consequently, health plans in Arizona may have care management expertise that minimizes the added value of D-SNP regulations. We also do not see beneficial effects of D-SNP regulation implementation in Tennessee. This finding may reflect incomplete D-SNP integration requirements (only binding for D-SNPs developed after 2014). Conversely, we do see evidence of benefit of D-SNP regulations for hospitalization reductions in New Jersey, a state that did not have any integrated Medicare/Medicaid plans during the baseline period but that subsequently mandated heightened integration for All D-SNPs in the state.

Our finding of stronger D-SNP regulation effects on hospital utilization with increasing co-morbidity is particularly important because studies suggest that individuals with higher levels of co-morbidity are less likely to enroll in managed care plans and are more likely to leave these plans even in states with seamless conversion [22]. Consequently, D-SNPs may be limited in in the hospital utilization and healthcare costs reductions they can achieve. In order to realize the full potential of D-SNPs to impact hospitalizations and healthcare costs, it will be important for D-SNPs to develop an understanding of the factors underlying enrollment decisions among dual-eligible beneficiaries with high levels of co-morbidity and to use this information to develop targeted outreach to this population [23]. One study found that concerns about service restrictions and loss of access to trusted providers may be important drivers of enrollment decisions [24]. Furthermore, implementing a pro-active strategy to retain this population once they have been enrolled will be important.

Lastly, we do not find differential effects of D-SNP regulations among racial/ethnic minority groups. This finding is surprising given the higher baseline levels of ER/hospital utilization among racial/ethnic minorities, relative to non-minorities, in this sample (Table 2). One potential explanation for the null findings is that social, economic and contextual factors, not addressed by D-SNP regulations, are prominent drivers of disproportionate ER/hospital utilization among racial/ethnic minorities, enrolled in D-SNPs. The high prevalence of social factors that have been positively linked with ER and hospital utilization such as poverty, food insecurity, housing instability, limited access to transportation and low health literacy have been well-documented among D-SNP enrollees [25]. A recent qualitative analysis of high performing managed care plans found that along with improving care integration and coordination, these plans took additional steps to address social factors. Some of the strategies adopted by these plans included the provision of transportation and contractual agreements with community-based organizations to address social needs such as housing instability and food insecurity [26].

This study has important limitations. First, we cannot guarantee that our treatment and comparison groups would have followed constant outcome trends in the absence of the policy change; which would represent a violation of a key assumption of our study design. However, data limitations preclude us from using an alternative comparison group for this analysis. Additionally, our study does not adjust for selection bias. Since the treatment group reflects individuals who decided to enroll in D-SNPs, the findings of this study may not be attributable to the broader population of dual-eligible beneficiaries. However, we try to minimize this bias by controlling for a number of demographic, socioeconomic and health factors that have been associated with plan selection and ER/hospital utilization. Additionally, our study design does not control for interventions that are temporally proximate to the implementation of D-SNP regulations if they did not impact the ER/hospital utilization of both D-SNP and MMC enrollees in the same way. Data limitations also prevent us from evaluating the effects of D-SNP regulations on outcomes outside of ER/hospital utilization such as nursing home days which would be valuable for constructing a more comprehensive assessment of the impact of D-SNP regulations. Our study sample also comes from a single insurer and includes states with longstanding experience in implementing integrated care plans and that have been at the forefront of the managed care movement among dual-eligible beneficiaries. All D-SNPs in our analysis effectively operate as Highly Integrated D-SNPs (HIDE-SNPs) in that they offer Medicare and MLTSS or behavioral health benefits under a single plan, through a companion plan or through a subsidiary offered by the parent organization [27]. Consequently, the results of this study may not be reproducible among a broader cross-section of insurers and/or states. Nonetheless, these results may offer a bit of insight into what can be expected from the enhanced D-SNP integration requirements imposed by the 2018 Balanced Budget Act (BBA) which take effect in 2021. The 2018 BBA permanently authorizes D-SNPs but requires that they either operate as a Fully Integrated D-SNP (offer Medicare and MLTSS benefits under a single contract), HIDE-SNP or that they have a process for sharing hospital or SNP admission data with either the state or a state designee such as an MCO [27].

## Conclusion

While our study did not find an effect of D-SNP regulations on ER/hospital utilization among the broader population of dual-eligible beneficiaries, our findings suggest positive impacts of D-SNP regulations on hospitalizations in states with low baseline levels of integration that mandate integration across all D-SNPs. Additionally, the reduction in hospitalizations appears proportional to the co-morbidity level of the patient population. Consequently, the impact of D-SNPs on healthcare utilization and costs may be enhanced by increasing enrollment and retention among the beneficiaries with the highest level of co-morbidity in the most integrated plans.

## Supplementary Information

**Additional file 1.**

## Data Availability

The authors confirm that the data supporting the findings of this study are not available due to a data use agreement but can be requested from Dr. Donna O’Shea at UnitedHealthcare at (877) 842–3210.

## Abbreviations

D-SNPs: Dual-Eligible Special Needs Plans
MOC: Model of Care requirements
MIPPA: Medicare Improvement Act for Patients and Providers
PP-ACA: Patient Protection and Affordable Care Act
MMC: Medicaid Managed Care
NCQA: National Committee of Quality Assurance
ER: Emergency Room
MLTSS: Medicaid Managed Long Term Services and Supports
MITS: Multiple-group Interrupted Time Series
UHC: UnitedHealthcare
SSI: Supplemental Security Income
LPM: Linear Probability Model
ALTCS: Arizona Long Term Care System
