# Supplementary material for: The impact of Dual Eligible Special Need Plan regulations on healthcare utilization
Source: BMC Health Serv Res. 2021 Mar 7;21:206. doi: 10.1186/s12913-021-06228-3 (PMC7938466; doi:10.1186/s12913-021-06228-3)
Supplement: Supplementary file 1 — Additional file 1. [file 12913_2021_6228_MOESM1_ESM.docx]

The Impact of Dual Eligible Special Need Plan Regulations on Healthcare Utilization

Running Title: D-SNP Regulations and Care Utilization

1. Kimberly Danae Cauley Narain, MD, PhD, MPH^1,2^
2. Jessica Harwood, MS^1^
3. Carol Mangione, MD, MSPH^1,3^
4. O. Kenrik Duru, MD, MSHS^1^
5. Susan Ettner, PhD^1,3^

1. Division of General Internal Medicine and Health Services Research

Department of Medicine, University of California Los Angeles, 1100 Glendon Ave., Suite 850

Los Angeles, CA 90024

2. Center for Health Advancement, Fielding School of Public Health, University of California Los Angeles, 650 Charles Young Dr., 31-269 CHS Box 951772

Los Angeles, CA, 90095-1772

3. Health Policy and Management, Fielding School of Public Health, UCLA, 650 Charles Young Dr. S., 31-269 CHS Box 951772, Los Angeles, CA, 90095-1772

Corresponding Author

Name: Kimberly Danae Cauley Narain

Mailing Address: UCLA Division of General Internal Medicine and Health Services

Research (GIM/HSR)

1100 Glendon Ave., Suite 850

Los Angeles, CA 90024

Phone Number: (310) 794-2829

Fax Number: (310) 794-9824

Email: [KNarain@mednet.ucla.edu](mailto:KNarain@mednet.ucla.edu)

**Supplementary Table 1**

|  |  | **Transition Period (vs. Pre Period)** | | | | **Post Period (vs. Pre Period)** | | | |
| --- | --- | --- | --- | --- | --- | --- | --- | --- | --- |
| **Sample** | **Outcome** | **DID Level^A^** | **P-Value** | **DID Slope^B^** | **P-Value** | **DID Level^A^** | **P-Value** | **DID Slope^B^** | **P-Value** |
| Total^1^ | Any Hospitalization | -0.45% | 0.19 | -0.02% | 0.58 | -0.88% | 0.26 | -0.02% | 0.42 |
|  | Any ED Visit | -0.65% | 0.13 | 0.00% | 0.99 | -0.73% | 0.44 | 0.01% | 0.74 |
| Non-Hispanic White^2^ | Any Hospitalization | -0.73% | 0.101 | -0.01% | 0.80 | -1.55% | 0.144 | -0.03% | 0.41 |
|  | Any ED Visit | -0.61% | 0.27 | 0.03% | 0.53 | -0.29% | 0.824 | -0.01% | 0.77 |
|  |  |  |  |  |  |  |  |  |  |
| Non-Hispanic Black^3^ | Any Hospitalization | -0.59% | 0.51 | -0.02% | 0.80 | 0.86% | 0.66 | -0.08% | 0.22 |
|  | Any ED Visit | -0.47% | 0.66 | -0.09% | 0.17 | -1.26% | 0.54 | -0.01% | 0.90 |
|  |  |  |  |  |  |  |  |  |  |
| Hispanic^4^ | Any Hospitalization | -0.81% | 0.31 | -0.03% | 0.67 | -1.85% | 0.25 | 0.03% | 0.69 |
|  | Any ED Visit | -0.91% | 0.38 | 0.04% | 0.67 | -1.81% | 0.42 | 0.11% | 0.17 |
|  |  |  |  |  |  |  |  |  |  |
| Notes: Linear regression used for utilization outcomes. Sample is person-months from 2010-2015. *denotes significance at p < .05. Regression covariates include Group (treatment vs. comparator); a linear monthly time trend, indicators and splines for both the transition (2012-2013) and post periods (2014-2015); and the interactions between these variables. Other covariates included sex, age, race (full sample model only), indicator for SSI status, 18 comorbidity indicators, and state fixed effects. Repeated measures adjusted for using Huber-White robust standard errors, clustering at the person level. 1. N=360,405 2. N=205,371 3.N=90,506 4. N=64,528 | | | | | | | | | |
| A. Difference between treatment vs. comparator in the discontinuity (change in level) for the given period, measured using the interaction between Group & an indicator variable for the given period. | | | | | | | | | |
| B. Difference between treatment vs. comparator in the change in slope for the given period, measured using the interaction between Group & a spline variable for the given period. | | | | | | | | | |

*Interrupted time series (ITS) segmented regression analysis: difference-in-difference (DID) estimates, comparing treatment (dual-eligibles 65+ enrolled in a D-SNP) vs. comparator (individuals 60-64 enrolled in Medicaid Managed Care) on changes in monthly utilization time trends associated with DSNP Regulations*
